# Burden of disease-specific late effects after proton beam therapy in pediatric solid and CNS tumor survivors: a single-institution survey in Japan

**DOI:** 10.1093/jjco/hyag081

**Published:** 2026-05-19

**Authors:** Hiroko Fukushima, Sho Hosaka, Ryoko Suzuki, Yuni Yamaki, Masako Inaba, Kumie Nagatomo, Ai Muroi, Kouji Masumoto, Takashi Saito, Masashi Mizumoto, Hideyuki Sakurai, Hidetoshi Takada

**Affiliations:** Department of Pediatrics, University of Tsukuba Hospital, 2-1-1 Amakubo, Tsukuba, Ibaraki 305-8576, Japan; Department of Child Health, Institute of Medicine, University of Tsukuba, 1-1-1 Tennodai, Tsukuba, Ibaraki 305-8575, Japan; Department of Pediatrics, University of Tsukuba Hospital, 2-1-1 Amakubo, Tsukuba, Ibaraki 305-8576, Japan; Department of Child Health, Institute of Medicine, University of Tsukuba, 1-1-1 Tennodai, Tsukuba, Ibaraki 305-8575, Japan; Department of Pediatrics, University of Tsukuba Hospital, 2-1-1 Amakubo, Tsukuba, Ibaraki 305-8576, Japan; Department of Child Health, Institute of Medicine, University of Tsukuba, 1-1-1 Tennodai, Tsukuba, Ibaraki 305-8575, Japan; Department of Pediatrics, University of Tsukuba Hospital, 2-1-1 Amakubo, Tsukuba, Ibaraki 305-8576, Japan; Department of Pediatrics, University of Tsukuba Hospital, 2-1-1 Amakubo, Tsukuba, Ibaraki 305-8576, Japan; Department of Pediatrics, University of Tsukuba Hospital, 2-1-1 Amakubo, Tsukuba, Ibaraki 305-8576, Japan; Department of Neurosurgery, University of Tsukuba Hospital, 2-1-1 Amakubo, Tsukuba, Ibaraki 305-8576, Japan; Department of Pediatric Surgery, University of Tsukuba Hospital, 2-1-1 Amakubo, Tsukuba, Ibaraki 305-8576, Japan; Department of Radiation Oncology, University of Tsukuba Hospital, 2-1-1 Amakubo, Tsukuba, Ibaraki 305-8576, Japan; Department of Radiation Oncology, University of Tsukuba Hospital, 2-1-1 Amakubo, Tsukuba, Ibaraki 305-8576, Japan; Department of Radiation Oncology, University of Tsukuba Hospital, 2-1-1 Amakubo, Tsukuba, Ibaraki 305-8576, Japan; Department of Pediatrics, University of Tsukuba Hospital, 2-1-1 Amakubo, Tsukuba, Ibaraki 305-8576, Japan; Department of Child Health, Institute of Medicine, University of Tsukuba, 1-1-1 Tennodai, Tsukuba, Ibaraki 305-8575, Japan

**Keywords:** proton beam therapy, childhood cancer survivor, comorbidity, disease-specific comorbidity, skeletal abnormality

## Abstract

**Background:**

Proton beam therapy (PBT) has been increasingly adopted in pediatric oncology to reduce radiation-related toxicity; however, its long-term comorbidities remain incompletely understood. Because tumor types and treatment patterns often overlap across diagnostic groups, disease-specific evaluation may help clarify late effects following PBT.

**Methods:**

A mailed questionnaire survey was conducted between 2015 and 2021 among 304 pediatric cancer survivors who had received PBT at the University of Tsukuba Hospital between 1984 and 2020. Responses from 110 patients diagnosed before the age of 20 years and alive at the time of the survey were analyzed. A non-PBT cohort treated between 1976 and 2013 served as a comparison group.

**Results:**

The median follow-up duration was 6.3 years (range, 0.4–34.6). Thirty-six patients (33%) reported no comorbidities. Severe (grade ≥3) comorbidities occurred in 3 PBT patients (gait disturbance, renal failure requiring dialysis, and hemianopsia) and 1 non-PBT patient (secondary colorectal cancer that might have been avoided with PBT). Skeletal abnormality was the most frequent late effect, followed by hair abnormality, growth retardation, chronic skin disorders, endocrine dysfunction, dental abnormalities, and neurocognitive impairment. Skeletal abnormality was significantly associated with rhabdomyosarcoma and Ewing sarcoma family of tumors, whereas hormone replacement therapy was more common among patients with central nervous system germ cell tumors.

**Conclusions:**

This single-institution disease-specific analysis revealed distinct comorbidity patterns among childhood cancer survivors treated with PBT. Although severe late effects were rare, musculoskeletal and endocrine disorders were frequent, underscoring the need for diagnosis-tailored, long-term follow-up strategies.

## Teaser

This single-institution Japanese questionnaire-based study found distinct disease-specific late-effect patterns after pediatric proton therapy; severe events were rare, but musculoskeletal and endocrine complications were frequent.

## Introduction

The prognosis of pediatric cancer has markedly improved, with ~80% of patients now achieving long-term survival [1]. However, late effects among long-term survivors have become increasingly recognized in recent years [2–4]. The age at diagnosis, tumor site, chemotherapy regimen, radiation dose and modality, and surgical approach often overlap or show common patterns among specific diagnostic groups of childhood cancers. Accordingly, several studies examining long-term late effects among childhood cancer survivors (CCSs) have analyzed their data according to diagnostic categories [2].

Radiotherapy plays an important role in the treatment of pediatric solid and brain tumors, and a considerable number of children undergo radiotherapy each year. Proton beam therapy (PBT) has been increasingly introduced as a modern radiotherapy modality; however, its availability remains limited, and its long-term effects are not yet fully understood. We performed a disease-specific analysis of late comorbidities in pediatric solid and brain tumor survivors who had received PBT.

## Methods

### Patients and data collection

We performed a mailed questionnaire survey between 2015 and 2021 among pediatric cancer patients who had received PBT at the University of Tsukuba Hospital between 1984 and 2020. Eligible participants were those diagnosed before 20 years of age and without any record of death or terminal illness in their medical charts at the time of the survey. Questionnaires were sent to 304 patients, and responses from 110 individuals were included in the analysis. For participants who submitted multiple responses, data from the most recent survey were used.

As a comparison group, we used results from a survey conducted in 2021 among pediatric solid and brain tumor patients treated at the University of Tsukuba Hospital between 1976 and 2013 who had not received PBT. Clinical background information was retrospectively obtained from the medical records. Current comorbidities were evaluated on the basis of the questionnaire (Supplementary document 1) responses and graded according to the Common Terminology Criteria for Adverse Events (CTCAE), version 5.0.

### Statistical analysis

Comparisons of comorbidity prevalence by diagnostic category among pediatric solid and brain tumor patients treated with PBT were also analyzed by use of the chi-square or Fisher’s exact test. Additional chi-square tests were conducted to compare the prevalence of hormone replacement therapy between the central nervous system germ cell tumor (CNS-GCT) and non–CNS-GCT groups and the prevalence of chronic skin disorders between the solid tumor and brain tumor groups. Analyses were stratified by disease group (solid tumors, CNS tumors) and by radiotherapy modalities (PBT or non-PBT). Between-group comparisons of the background characteristics and comorbidities of the PBT and non-PBT groups were conducted by use of the chi-square test or Fisher’s exact test, as appropriate. Differences in the maximum CTCAE grade per individual were evaluated by use of the Mann–Whitney *U* test. The health-related quality of life results and CTCAE grade distributions in the PBT and non-PBT survivors from this cohort have been reported previously [5].

### Ethical statement

This study was approved by the ethics committee of the University of Tsukuba Hospital (approval number: H27–137) and conducted in accordance with the Ethical Guidelines for Medical Research Involving Human Subjects issued by the Ministry of Health, Labour and Welfare of Japan and with the principles of the Declaration of Helsinki. Written informed consent was obtained from all the patients and/or their guardians before participation.

## Results

**Figure 1:**
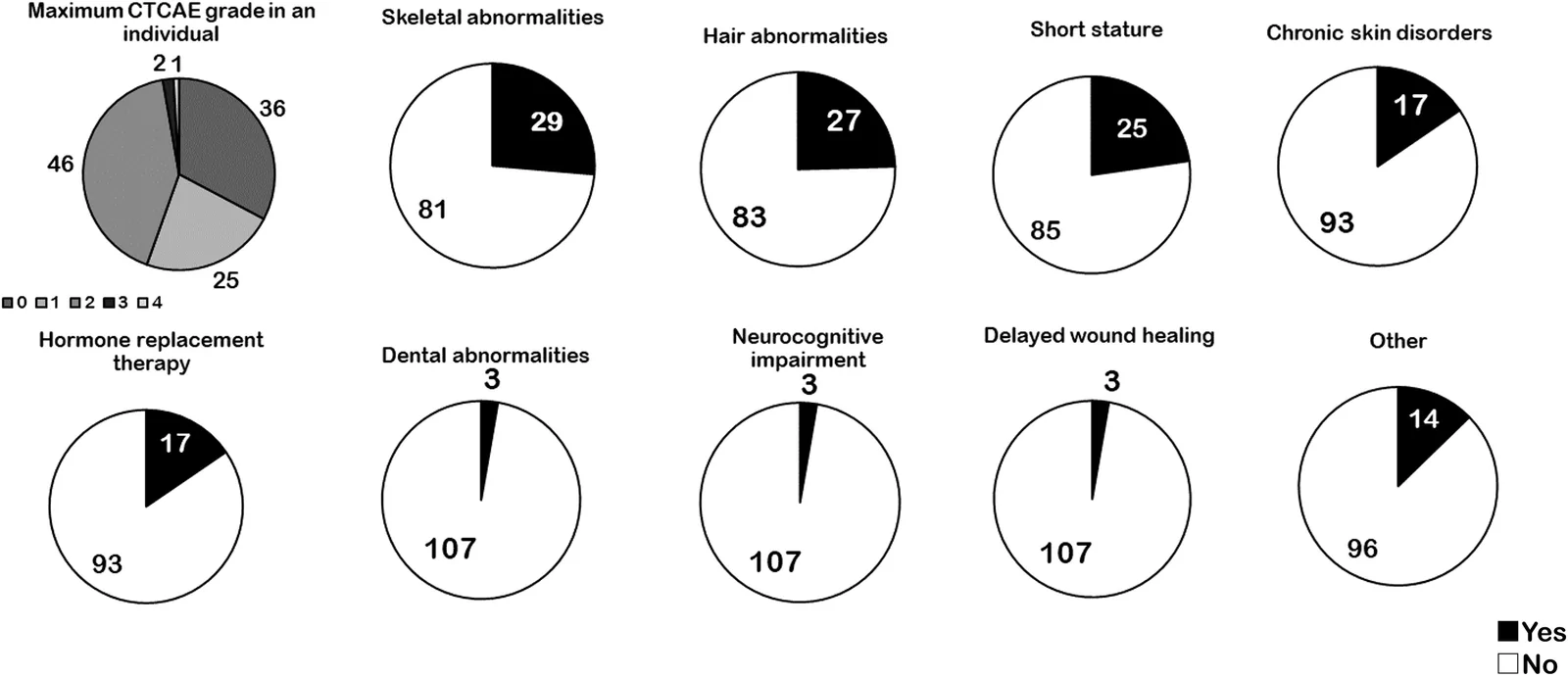
Comorbidity that occurs in CCSs treated with PBT. The upper left panel shows the maximum CTCAE grade observed in each individual. Severe comorbidities (grade ≥3) were observed in three patients: Gait disturbance, renal failure requiring dialysis, and hemianopsia necessitating the use of a white cane. The other panels indicate the presence (black) or absence (white) of each comorbidity among all the patients who received PBT.

A total of 110 valid responses from pediatric cancer survivors treated with PBT were analyzed. Fifty-eight of the patients (53%) were male (Table 1). The median age at diagnosis was 6.7 years (range, 0.4–15.9); the median age at the time of survey, 13.2 years (range, 7.0–37.8); and the median follow-up duration, 6.3 years (range, 0.4–34.6). Thirty-eight patients had CNS tumors, and 72 had solid tumors. The diagnostic distribution of these patients categorized as having other solid tumors or CNS tumors are summarized in Supplementary Table S1. The most common comorbidity was skeletal abnormalities, which was followed by hair abnormality, short stature, skin disorders, hormone replacement therapy, dental abnormalities, neurocognitive dysfunction, and delayed wound healing (Fig. 1). Details of the other types of comorbidities are provided in Supplementary Table S2. The distribution of the highest CTCAE grade for each patient is shown in Fig. 1 (left panel). Severe comorbidities (grade ≥3) were observed in only three patients: gait disturbance, renal failure requiring dialysis, and hemianopsia requiring the use of a white cane.

**Table 1:** Patient characteristics.

		**All**		**Central nervous system**			**Solid tumors**	
	*n*	110			38			72
	Male	58			24			34
Age at diagnosis or radiation therapy, y	Median	6.7			8.7			5.5
	(Range)	(0.4–15.9)			(1.8–15.9)			(0.4–14.8)
Age at survey, y	Median	13.2			14.3			12.8
	(Range)	(7.0–37.8)			(8.1–24.8)			(7.0–37.8)
Years from diagnosis or radiation therapy	Median	6.3			5.7			5.3
	(Range)	(0.4–34.6)			(0.4–12.2)			(0.4–34.6)
Radiotherapy, yes		110			38			72
Dose of radiotherapy, Gy	Median	50.4			50.4			50.4
	(Range)	(10.8–78.4)			(24–78.4)			(10.8–75)
Treatment era	1980–99	3			0			3
	2000–9	10			1			9
	2010–20	97			37			60
Diagnosis				CNS-GCT	15		Rhabdomyosarcoma	29
				Ependymoma	9		ESFT	16
				Medulloblastoma	5		Neuroblastoma	11
				Malignant glioma	3		Others	16
				cPNET	2			
				Others	4			

Abbreviations: ESFT, Ewing sarcoma family of tumors; CNS-GCT, central nervous system-germ cell tumors; cPNET, central primitive neuroectodermal tumor.

### Analysis by disease diagnostic group

**Figure 2:**
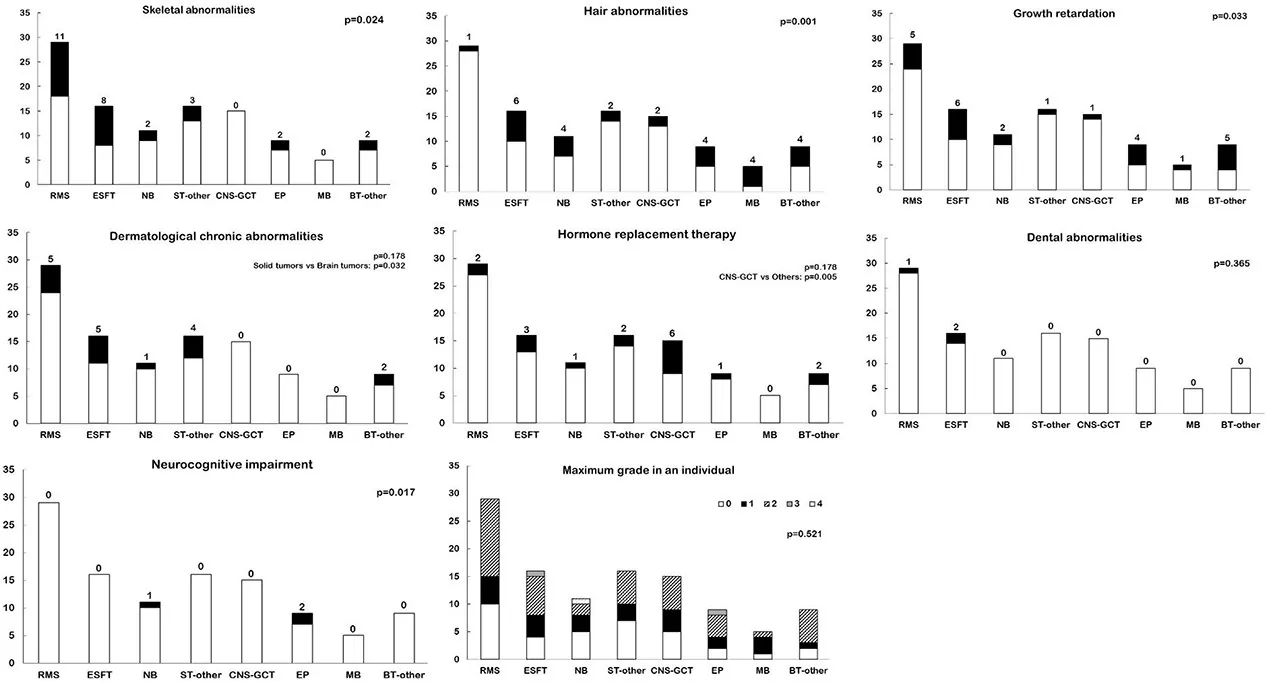
Comorbidity profiles according to diagnostic group. Each bar indicates the number of cases with (black) or without (white) comorbidities in each diagnostic group. Abbreviations: RMS, rhabdomyosarcoma; ESFT, Ewing sarcoma family of tumors; NB, neuroblastoma; ST, solid tumor; CNS-GCT, central nervous system germ cell tumor; EP, ependymoma; MB, medulloblastoma; BT, brain tumor.

Skeletal abnormalities were significantly more common among patients with rhabdomyosarcoma or Ewing sarcoma family of tumors (Fig. 2, *P* = .024). Hair abnormalities were frequent in patients with Ewing sarcoma family of tumors (ESFT), neuroblastoma, or brain tumors, suggesting an association with cranial irradiation (*P* = .001). Growth retardation was observed mainly in patients with rhabdomyosarcoma, ESFT, ependymoma, or other brain tumors (*P* = .033). Skin disorders were more frequent in solid tumor patients than in brain tumor patients (*P* = .032). Hormone replacement therapy was more common in CNS-GCT (*P* = .005). Dental abnormalities were observed in patients with rhabdomyosarcoma or ESFT, but without significant intergroup differences (*P* = .365). Neurocognitive impairment was identified among patients with ependymoma or neuroblastoma. Nevertheless, the prevalence may have been underestimated, particularly in CNS tumors, because patients with severe cognitive dysfunction were less likely to respond to the questionnaire. Severe (grade ≥3) comorbidities were observed among patients with ESFT, ependymoma, or neuroblastoma, but no significant intergroup differences were found (*P* = .521).

### Analysis of tumor location and its laterality

**Figure 3:**
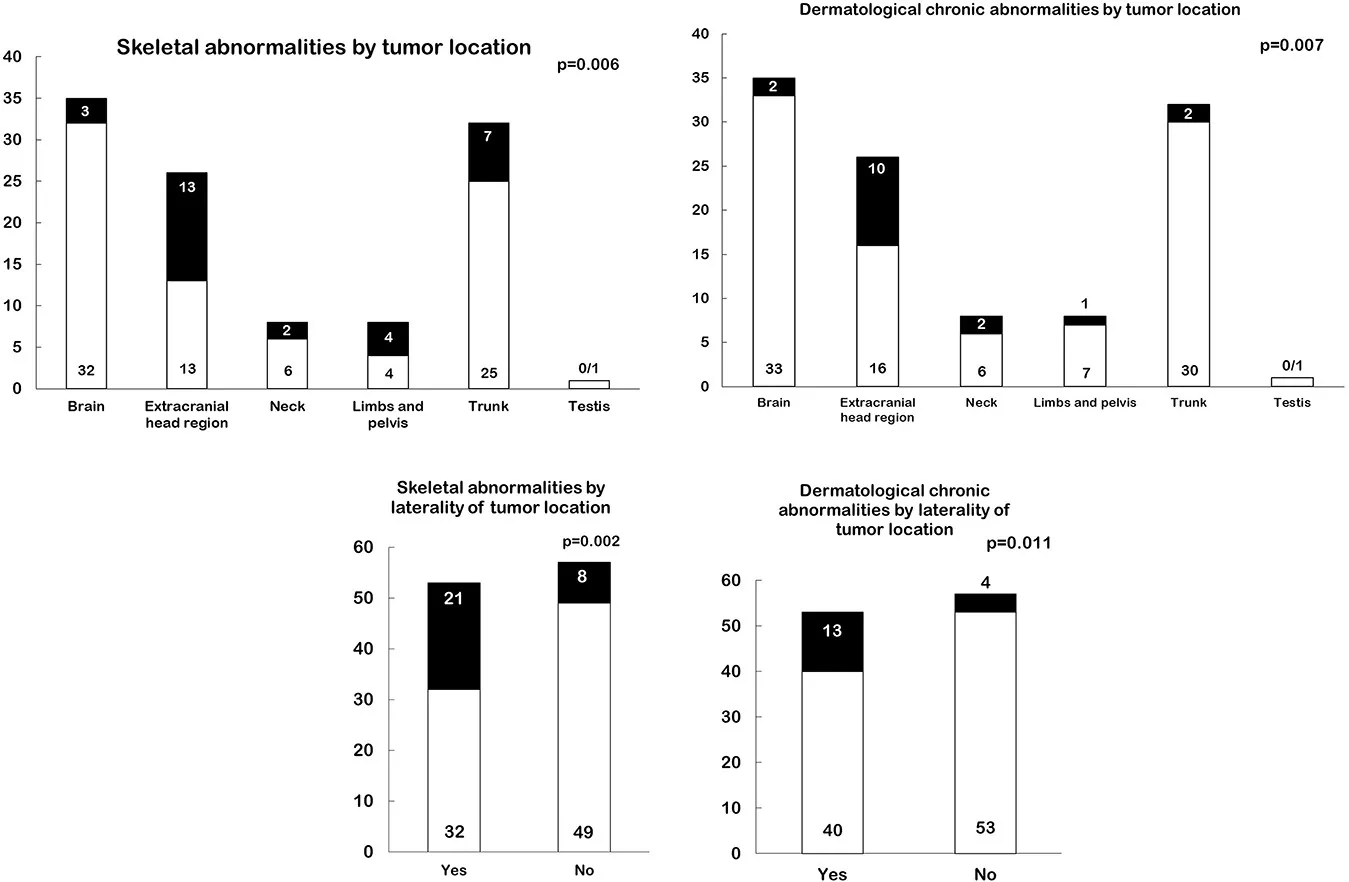
Skeletal abnormalities and dermatological chronic abnormalities by tumor location. Each bar indicates the number of cases with (black) or without (white) comorbidities in each tumor location.

Among the comorbidities observed, skeletal abnormalities and chronic skin chronic abnormalities were common. To further characterize these conditions, additional analyses were performed focusing on tumor location and laterality, assuming that such impairments might be more noticeable when associated with asymmetric or localized tumor sites. Skeletal abnormality was more frequently identified in patients with tumors located in the extracranial head region or in the limbs and pelvis (Fig. 3; *P* = .006). Furthermore, although pediatric tumors often arise in the midline, these comorbidities were more commonly observed when the tumor was lateralized to 1 side rather than located at the midline (*P* = .002 and *P* = .011, respectively).

### Additional comparison analysis between PBT and non-PBT groups

The patient backgrounds, including age at diagnosis and gender distribution, were comparable between the two groups, although the non-PBT cohort had an older median age at survey and a longer follow-up duration (Supplementary Table S3). In the non-PBT group, 1 patient experienced a grade ≥3 event—colorectal cancer. Although it is unclear whether this patient had a hereditary tumor predisposition, the colorectal cancer arose adjacent to the previous irradiation field. The patient received treatment in the early 1990s; if modern PBT had been used, the radiation exposure to the portion of the intestinal tract could have been reduced (Supplementary Fig. S1). The patient later died from this subsequent malignancy. Maximum CTCAE grade and type of comorbidities were comparable between the PBT and non-PBT groups (Supplementary Fig. S2).

## Discussion

We conducted a disease-specific analysis of long-term comorbidities among pediatric cancer survivors who had received PBT. Although previous reports have described late effects by individual diagnoses such as medulloblastoma treated with PBT, to our knowledge, no previous studies have compared comorbidities across diagnostic categories. A nationwide Japanese survey previously reported grade ≥3 late effects in ~17% of pediatric cancer survivors, a higher rate than in our study [6]. This discrepancy likely reflects the nature of our questionnaire-based design, which may have resulted in lower response rates among patients with severe sequelae. The most commonly reported comorbidities in previous studies—hair abnormalities and skeletal deformities—were consistent with our findings.

Several studies have also investigated outcomes after PBT therapy for medulloblastoma, reporting that patients who underwent PBT showed significantly better full-scale IQ (FSIQ) outcomes [7, 8]. A meta-analysis further concluded that, among pediatric brain tumor patients, PBT reduces late toxicity without increasing tumor recurrence [7–10].

Disease-specific comorbidity profiles among pediatric cancer survivors—regardless of radiation modality—have also been reported from the St. Jude Lifetime Cohort Study [2]. In our study, the highest CTCAE grade per patient did not differ significantly among the diagnostic categories. Previous reports have shown a slightly higher rate of comorbidities among CNS tumor survivors and a slightly lower rate among Wilms tumor survivors, findings consistent with ours [2].

Skeletal abnormalities were particularly common among patients with rhabdomyosarcoma or ESFT. These tumors often arise in non-midline regions, and asymmetric skeletal growth due to localized high-dose irradiation (~50 Gy) may become apparent. Additionally, the frequency of skeletal abnormalities and chronic skin disorders varied according to whether the primary tumor was located at the midline or in a lateralized organ (Fig. 3). These findings indicate that the likelihood and detectability of such comorbidities may depend on the anatomical site of the tumor. These observations underscore the importance of considering the potential impact of tumor location on the development and recognition of late complications, particularly during initial treatment planning for very young patients whose bodies are still undergoing growth and maturation. Hair abnormalities were observed mainly among patients with brain tumors, ESFT, or neuroblastoma, likely reflecting cranial irradiation or, in the case of neuroblastoma, high-dose chemotherapy with busulfan. Growth retardation was frequent among ESFT and CNS patients, possibly influenced by craniospinal irradiation and younger treatment age. Hormone replacement therapy was most frequent among CNS-GCT survivors, consistent with previous reports showing high involvement of the hypothalamic–pituitary axis in such cases [11]. The prevalence of neurocognitive impairment was lower than those reported in previous studies [7, 9], likely reflecting the limitations of a self-administered questionnaire, as patients with neurological deficits may have been less likely to respond.

In this study, we compared the late effects between CCSs who received PBT and those who did not. Although the PBT and non-PBT groups did not differ in age at diagnosis, substantial differences were found in treatment era, the proportion of patients who received radiotherapy, radiation dose, follow-up duration, and age at the time of the survey (Supplementary Table S3). Previous studies have reported that CCSs who received radiotherapy or who have longer follow-up periods tend to develop more late complications [2, 3]. Therefore, the results of comparative analyses should be interpreted with caution. Nevertheless, the individual CTCAE grades were comparable between the two groups, and the prevalence of each comorbidity did not differ markedly. Future studies comparing survivors with similar ages and follow-up durations will be essential to more accurately evaluate the long-term adverse effects associated with PBT.

This study has several limitations. First, the questionnaire-based design may have introduced selection bias, with responses more likely from healthier survivors. Consequently, the prevalence of cognitive or neurological impairment may have been underestimated. Second, as a single-institution study, the distribution of cancer diagnoses and treatment practices in our cohort may differ from those seen in the general pediatric cancer population. Third, the non-PBT group had a longer follow-up period and was older at the time of the survey than the PBT group, which may have affected the spectrum and frequency of comorbidities observed. Furthermore, the non-PBT group was treated in earlier eras, during which treatment protocols, therapeutic approaches, and supportive care standards likely differed from those currently in use. Fourth, the sample size of our cohort is relatively small; therefore, the findings may not be generalizable to the broader population.

In conclusion, we conducted the first disease-specific analysis of late comorbidities among pediatric cancer survivors treated with PBT. Our cohort included patients with follow-up durations exceeding 34 years. These findings provide valuable insights into long-term health risks for CCSs treated with PBT and may inform future risk prediction models when integrated with large-scale, modality-independent cohort data. In particular, bone growth impairment was more pronounced among patients who received irradiation to the extracranial head region or to the extremities and pelvis. This finding provides important insight for predicting future skeletal complications.

## Supplementary Material

CCS_Comorbid_SF1_20251113_hyag081

CCS_Comorbid_SF2_20251031_hyag081

CCS_Comorbid_ST1_20260414_hyag081

CCS_Comorbid_ST2_20260305_hyag081

CCS_Comorbid_ST3_20260305_hyag081

## Data Availability

Data will be made available from the authors upon reasonable request.

## Prior presentation

Parts of the contents of this manuscript were presented at the 63rd and 64th annual meetings of the Japanese Society of Pediatric Hematology/Oncology, web-based in 2021 and at Tokyo in 2022, and at the 128th annual meeting of the Japan Pediatric Society at Aichi in 2025, respectively.
