# Supplementary material for: Burden of disease-specific late effects after proton beam therapy in pediatric solid and CNS tumor survivors: a single-institution survey in Japan
Source: Jpn J Clin Oncol. 2026 May 19;56(9):1074–9. doi: 10.1093/jjco/hyag081 (PMC13556386; doi:10.1093/jjco/hyag081)
Supplement: Supplementary file 1 [file ccs_comorbid_st1_20260414_hyag081.docx]

Supplementary Table 1: Patient characteristics

		All			Central nervous system				Solid tumors			
		PBT	no-PBT	p-value		PBT	no-PBT	p-value		PBT	no-PBT	p-value
	n	110	21			38	9			72	12	
	Male	58	15	0.114		24	7	0.433		34	8	0.212
Age at diagnosis or radiation therapy, y	Median	6.7	5.3	1.000		8.7	9.2	0.457		5.5	4.6	0.510
	(range)	(0.4-15.9)	(0.0-14.4)			(1.8-15.9)	(1.8-14.2)			(0.4-14.8)	(0.0-14.4)	
Age at survey, y	Median	13.2	23.6	p<0.001†		14.3	26.3	<0.001†		12.8	20.8	<0.001†
	(range)	(7.0-37.8)	(17.0-36.2)			(8.1-24.8)	(19.2-32.2)			(7.0-37.8)	(17.0-36.2)	
Years from diagnosis or radiation therapy	Median	6.3	16.4	p<0.001†		5.7	15.6	<0.001†		5.3	17.3	0.013†
	(range)	(0.4-34.6)	(8.3-27.0)			(0.4-12.2)	(8.3-27.0)			(0.4-34.6)	(8.8-25.6)	
		110	13	p<0.001†		38	7	0.033†		72	7	<0.001†
	Median	50.4	10.0	p<0.001†		50.4	45	0.003†		50.4	0	<0.001†
	(range)	(10.8-78.4)	(0-50.4)			(24-78.4)	(0-50.4)			(10.8-75)	(0-50.4)	
	1980-1999	3	7	p<0.001†		0	3	p<0.001†		3	4	p<0.001†
	2000-2009	10	12			1	5			9	7	
	2010-2020	97	2			37	1			60	1	
Diagnosis					CNS-GCT	15	4	0.241	Rhabdomyosarcoma	29	4	0.120
					Ependymoma	9	0		ESFT	16	0	
					Medulloblastoma	5	3		Neuroblastoma	11	2	
					Malignant glioma	3	2		Others	16	6	
					cPNET	2	0					
					Others	4	0					

† means p<0.05. Abbreviations: ESFT, Ewing sarcoma family of tumors; CNS-GCT, central nervous system-germ cell tumors; cPNET, central primitive neuroectodermal tumor
