# Supplementary material for: Burden of disease-specific late effects after proton beam therapy in pediatric solid and CNS tumor survivors: a single-institution survey in Japan
Source: Jpn J Clin Oncol. 2026 May 19;56(9):1074–9. doi: 10.1093/jjco/hyag081 (PMC13556386; doi:10.1093/jjco/hyag081)
Supplement: Supplementary file 2 [file ccs_comorbid_st2_20260305_hyag081.docx]

| Supplementary Table 2: List of diagnostic distributions in other solid tumors or other CNS tumors | | |
| --- | --- | --- |
| Proton beam therapy | Nasopharyngeal carcinoma | 4 |
|  | Osteosarcoma | 2 |
|  | Retinoblastoma | 2 |
|  | Germ cell tumors | 2 |
|  | Renal clear cell carcinoma | 1 |
|  | Wilms tumor | 1 |
|  | Synovial sarcoma | 1 |
|  | Maxillary adenoid carcinoma | 1 |
|  | Cervical desmoid tumor | 1 |
|  | Parotid gland carcinoma | 1 |
| No proton beam therapy | Germ cell tumors | 2 |
|  | Wilms tumor | 1 |
|  | Hepatoblastoma | 1 |
|  | Pleuropulmonary blastoma | 1 |
|  | Adrenocortical carcinoma | 1 |
| Abbreviation: CNS: central nervous system | | |
