# Supplementary material for: Burden of disease-specific late effects after proton beam therapy in pediatric solid and CNS tumor survivors: a single-institution survey in Japan
Source: Jpn J Clin Oncol. 2026 May 19;56(9):1074–9. doi: 10.1093/jjco/hyag081 (PMC13556386; doi:10.1093/jjco/hyag081)
Supplement: Supplementary file 3 [file ccs_comorbid_st3_20260305_hyag081.docx]

| Supplementary Table 3: List of other comorbidities | |
| --- | --- |
| Proton beam therapy | Low body weight |
|  | Nausea |
|  | Reduced salivation |
|  | Asymmetry of muscle mass |
|  | Renal failure and peritoneal dialysis |
|  | Hemianopia |
|  | Epilepsy |
|  | Cerebral infarction |
|  | Inability to walk |
|  | Loss of sensation in lower limbs |
|  | Scoliosis |
|  | Easy fatigability |
|  | Peripheral neuropathy |
|  | Ataxic symptoms |
| No proton beam therapy | Colorectal cancer |
|  | Hypertension |
|  | Lifestyle-related disease |
|  | Visual impairment |
|  | Hearing impairment |
